# Preparation and Screening of SRB Gel Particles Used for Deep Purification of Acid Mine Drainage

**DOI:** 10.3390/molecules29133217

**Published:** 2024-07-06

**Authors:** Chunpeng Leng, Xi He, Yukuo Liu, Lifeng Shi, Fuping Li, Hao Wang, Cong Zhao, Siyu Yi, Lei Yu

**Affiliations:** 1 College of Mining Engineering, North China University of Science and Technology, Tangshan 063210, China; lengchunpeng@ncst.edu.cn; 2 Key Laboratory of Bioelectrochemical Water Pollution Control Technology in Tangshan City, North China University of Science and Technology, Tangshan 063210, Chinachris20010926@163.com (L.Y.); 3 Hebei Industrial Technology Institute of Mine Ecological Remediation, Tangshan 063210, China; 4 College of Civil and Architectural Engineering, North China University of Science and Technology, Tangshan 063210, China

**Keywords:** acid mine drainage, sulfate reducing bacteria, sulfate root removal rate, gel particles

## Abstract

The progressive decline of the coal industry necessitates the development of effective treatment solutions for acid mine drainage (AMD), which is characterized by high acidity and elevated concentrations of heavy metals. This study proposes an innovative approach leveraging sulfate-reducing bacteria (SRB) acclimated to contaminated anaerobic environments. The research focused on elucidating the physiological characteristics and optimal growth conditions of SRB, particularly in relation to the pH level and temperature. The experimental findings reveal that the SRB exhibited a sulfate removal rate of 88.86% at an optimal temperature of 30 °C. Additionally, SRB gel particles were formulated using sodium alginate (SA) and carboxymethyl cellulose (CMC), and their performance was assessed under specific conditions (pH = 6, C/S = 1.5, T = 30 °C, CMC = 4.5%, BSNa = 0.4 mol/L, and cross-linking time = 9 h). Under these conditions, the SRB gel particles demonstrated an enhanced sulfate removal efficiency of 91.6%. Thermal analysis via differential scanning calorimetry (DSC) and thermogravimetric analysis (TGA) provided further insights into the stability and properties of the SRB gel spheres. The findings underscore the potential of SRB-based bioremediation as a sustainable and efficient method for AMD treatment, offering a novel and environmentally friendly solution to mitigating the adverse effects of environmental contamination.

## 1. Introduction

In addition to the decline in the coal industry’s backward production capacity, a significant number of coal mines have closed. The closure of coal mines has resulted in a number of environmental issues, with acid mine drainage (AMD) being a significant concern [1,2,3]. It is imperative that the issue of effective management of AMD be addressed with the utmost urgency.

AMD is characterized by strong acidity, high hardness, and a high concentration of heavy metals [4,5,6]. Such contamination will not only affect surface water quality but will also have an impact on groundwater resources. As a consequence of the substantial influx of groundwater into the goaf water, precious water resources are transformed into mine wastewater, thereby resulting in a significant waste of groundwater resources [7,8,9].

Sulfate-reducing bacteria (SRB) are a class of anaerobic microorganisms that metabolize by using sulfate (SO_4_^2−^) as an electron acceptor. These bacteria are able to survive in anoxic or anaerobic environments and obtain energy by reducing sulfate to hydrogen sulfide (H_2_S). The wide distribution of sulfate-reducing bacteria in nature and their ecological and industrial importance have made them a focus of research. Recent studies have demonstrated that sulfate-reducing bacteria (SRB) can effectively treat acid mine water through inoculation, with the resulting treatment being referred to as AMD [10,11,12,13,14,15,16]. In the absence of oxygen, SRB reduce the sulfate in AMD to hydrogen sulfide, thereby reducing the sulfate content. Hydrogen sulfide continues to react with the metal ions in wastewater to form metal sulfide precipitation, which then removes the metal ions in AMD while producing alkaline substances to increase the pH level [17,18,19,20]. The creation of an optimal microenvironment for SRB and the immobilization of SRB gel particles are beneficial for enhancing the efficacy and stability of the treatment process [21,22,23].

In this study, wastewater samples from an area affected by AMD and the pretreatment process were collected. These samples were then used for the enrichment, culture, separation, and screening of SRB. A highly efficient strain was developed for the treatment of sulfate in AMD. Sodium alginate (SA) was employed to immobilize the SRB, while carboxymethyl cellulose (CMC) was used to enhance the mechanical properties of SA. This resulted in the formation of SRB gel particles through a cross-linking and embedding method. Furthermore, the physicochemical properties, electrical conductivity, water loss rate, and sulfate removal rate of the gel particles were investigated in order to provide experimental guidance for the advanced treatment of AMD.

## 2. Results and Discussion

This section may be divided into subheadings. The text should provide a succinct and accurate account of the experimental outcomes, their interpretation, and the conclusions that can be drawn from the experiment.

### 2.1. Preliminary Screening of Sulfate-Reducing Bacteria

**Figure 1:**
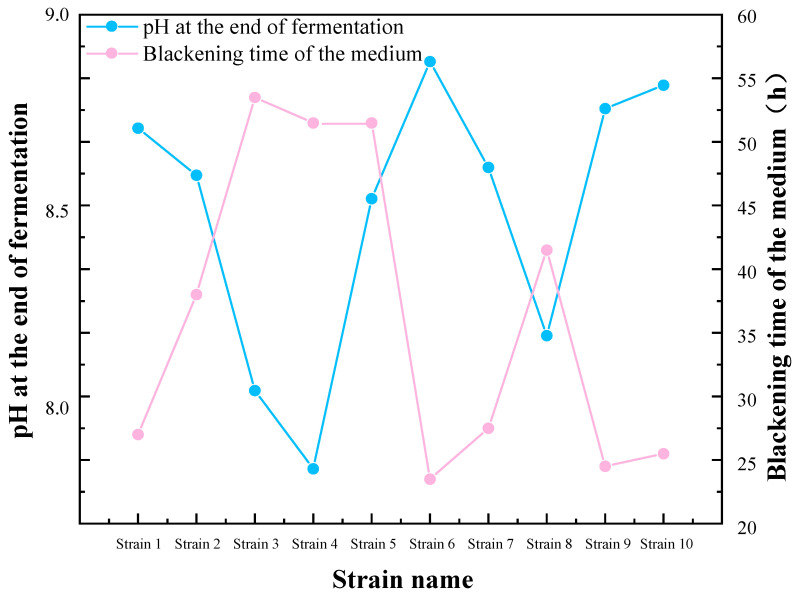
Preliminary screening and pH variations of sulfate-reducing bacteria (SRB) strains.

The preliminary screening results, as indicated in Table 1 and Figure 1, indicate that the media of strains 1, 6, 9, and 10 exhibited the fastest blackening, accompanied by a notable increase in pH at the end of the culture period. This suggests that these four strains exhibited a pronounced ability to reduce sulfate, and they were therefore selected for subsequent experiments. The four strains in the aforementioned preliminary screening were designated as SRB-1, SRB-6, SRB-9, and SRB-10.

The results of the aforementioned experimental procedure (Table 2) indicate that the four strains were all Gram-negative bacteria. All four strains were negative in the V-P test and were unable to utilize glucose. However, they were able to utilize sucrose and lactose. The strains SRB-1, SRB-6, and SRB-10 exhibited positive results in the gelatin liquefaction experiment, whereas the strain SRB-9 displayed negative results. The four SRB strains exhibit similarities with those reported by other scholars with regard to their physiological and biochemical characteristics.

### 2.2. Growth Curve and SO_4_^2−^ Reduction Effect of SRB Strain

**Figure 2:**
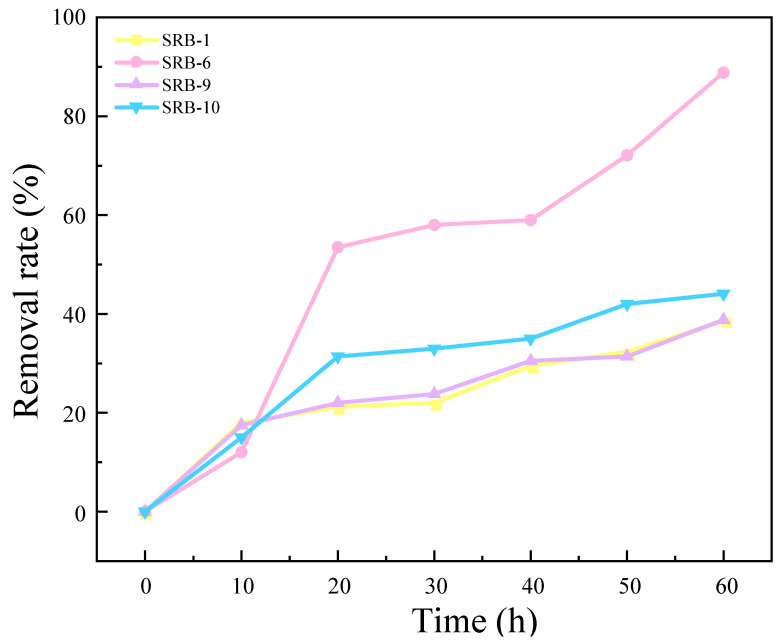
Sulfate removal rates for different SRB strains over time.

The results of the experimental investigation into the ability of four strains to remove SO_4_^2−^ are presented in Figure 2. The results demonstrate that SRB-1, SRB-6, SRB-9, and SRB-10 were capable of reducing SO_4_^2−^. Of these, SRB-6 exhibited the greatest reducing ability, reducing 88.75% of the SO_4_^2−^ in the medium over a 60 h period. The removal rates of SRB-1, SRB-9, and SRB-10 were 33.56%, 32.69%, and 46.05%, respectively, under the same culture time.

**Figure 3:**
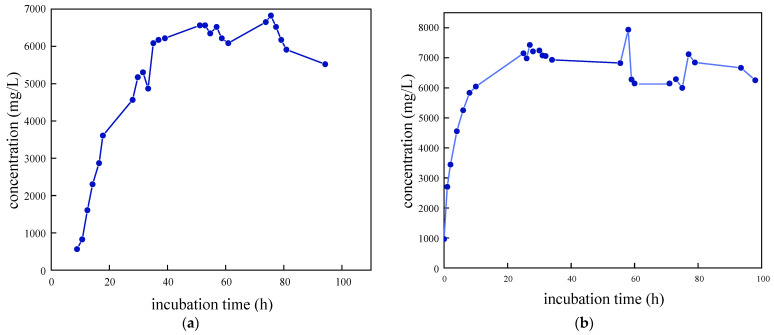

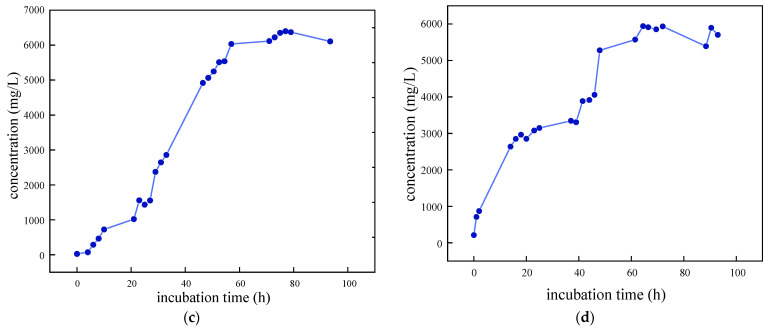
Growth characteristic curves of different SRB strains: (**a**) SRB-1; (**b**) SRB-6; (**c**) SRB-9; and (**d**) SRB-10.

As illustrated in Figure 3, the growth curves of the four SRB strains were determined and analyzed following preliminary screening. The removal rate of SO_4_^2−^ was also evaluated. It was observed that SRB-6 exhibited the shortest time to enter the logarithmic phase and demonstrated the most effective removal of SO_4_^2−^. Consequently, strain 6 was selected for further investigation.

### 2.3. Analysis of Physical and Chemical Properties of SRB Strains

**Figure 4:**
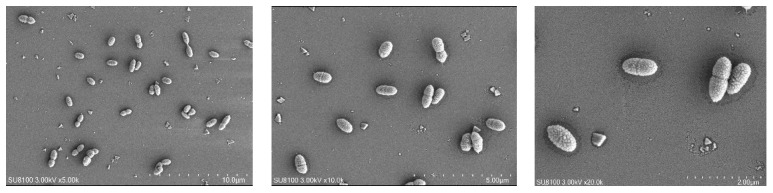
Scanning electron microscopy (SEM) images of SRB strains at different magnifications.

The cell morphology, cell size, and movement characteristics of SRB-6 were observed using scanning electron microscopy. The results are shown in Figure 4.

Through analysis of the morphology and growth characteristics of the selected SRB strains, it was found that the individuals were rod-shaped, singly arranged non-spores, and the colonies were black.

The rod-shaped morphology and single arrangement of SRB-6 are typical characteristics of many sulfate-reducing bacteria. These morphological features confer an advantage in terms of motility and interaction with the surrounding environment. The absence of spore formation indicates that SRB-6 relies on continuous metabolic activity for survival rather than entering a dormant state, which is a common strategy employed by other bacterial species to withstand unfavorable conditions.

The black coloration of the colonies is a consequence of the production of metal sulfides as a result of sulfate reduction. When SRB-6 reduces sulfate (SO_4_^2−^) to sulfide (S^2−^), the sulfide can react with metal ions present in the medium, forming black metal sulfide precipitates. This is a common indicator of active sulfate reduction and is frequently employed as a qualitative measure of SRB activity in cultures.

Consequently, the observed morphological and growth characteristics are consistent with the known metabolic functions of SRB-6, particularly its role in sulfate reduction and the subsequent formation of metal sulfide precipitates. These characteristics are fundamental to its ecological function and have the potential to be applied in the fields of bioremediation and bioenergy production.

### 2.4. The Effect of Different pH Values on the Reduction of SO_4_^2−^ by an SRB Strain

**Figure 5:**
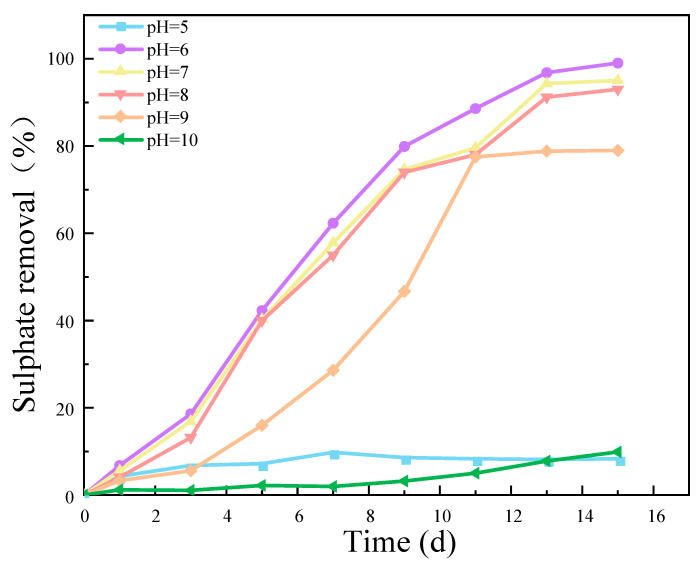
Effect of different pH values on SO_4_^2−^ removal rate. (The C/S was 1, the concentration of SO_4_^2−^ was 1400 mg/L, and the speed was 50 r/min).

The results presented in Figure 5 demonstrate that the removal efficiency of SO_4_^2−^ exhibited a pronounced dependence on the pH value. This indicates that the pH value is a crucial factor in determining the activity of an SRB strain. Both excessively low and high pH values had a detrimental impact on the activity of the SRB strains, thereby affecting the reduction of SO_4_^2−^. The results of this experiment demonstrate that when the pH level was between 6 and 7, the removal rate of SO_4_^2−^ was closest to 100%. This indicates that the SRB-6 strain, which was the subject of this experiment, is most suitable for growth and activity in a pH range from 6 to 7.

The optimal pH range for the removal of SO_4_^2−^ by SRB-6 was between 6 and 7. This is due to the fact that the enzymes involved in the sulfate reduction pathway exhibit optimal catalytic efficiency within this pH range. Furthermore, the stability and integrity of the bacterial cell membrane, which is essential for nutrient uptake and waste expulsion, were maintained best at this pH level. The pH range of 6–7 is conducive to the availability and solubility of essential nutrients and minerals required for bacterial growth and metabolism. Furthermore, extreme pH levels can result in the accumulation of toxic compounds that inhibit bacterial growth. Consequently, the pH range of 6–7 minimizes the toxic effects, thereby creating an optimal environment for SRB activity [24,25,26].

### 2.5. The Effect of Different Temperatures on the Reduction of SO_4_^2−^ by an SRB Strain

**Figure 6:**
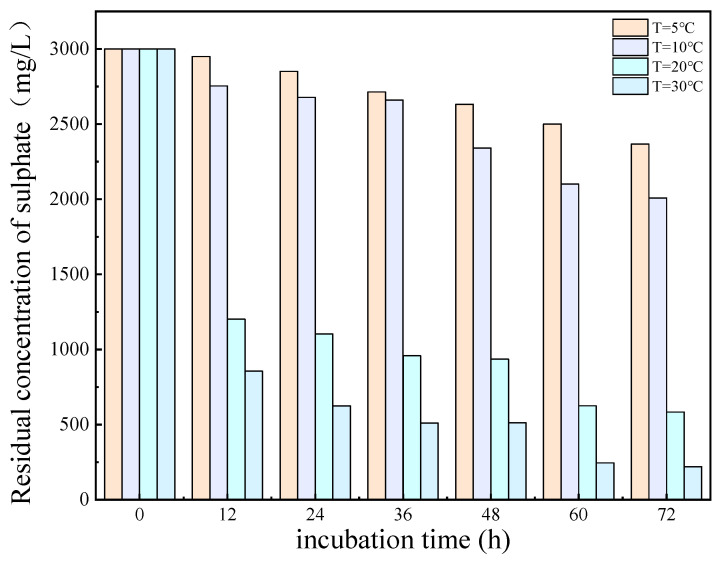
Residual concentration of sulfate (SO_4_^2−^) at different temperatures over incubation time.

**Figure 7:**
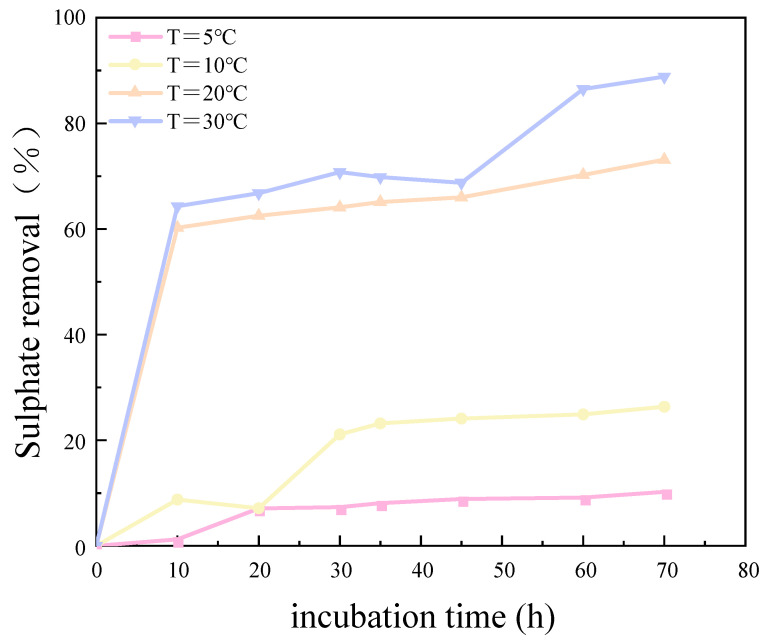
Sulfate removal rate at different temperatures over incubation time.

The results of the experimental investigation, presented in Figure 6 and Figure 7, demonstrate that the temperature exerted a significant influence on the growth and metabolic activity of microorganisms. The rate of removal of SO_4_^2−^ also varied with the temperature. At 5 °C, the metabolic activity of the SRB strain was inhibited, resulting in a reduction in the growth rate and a significantly diminished removal rate of SO_4_^2−^. In the temperature range of 10–30 °C, the growth rate of the strain increased with rising temperatures, resulting in an increased removal rate of SO_4_^2−^. At 30 °C, the SRB strain exhibited the highest removal efficiency for SO_4_^2−^.

The optimum temperature for SO_4_^2^ removal by SRB-6 was 30 °C, because the metabolic rate of the SRB strains generally increased with the temperature and reached peak efficiency at this point. At 30 °C, the enzymes and cellular processes involved in sulphate reduction operated at their best. In addition, higher temperatures within this optimum range enhanced essential cellular functions such as nutrient uptake, energy production, and waste removal, which are critical for sustained metabolic activity. This temperature also supported faster bacterial growth and proliferation, resulting in higher biomass amounts capable of reducing more sulphate.

The removal rates of SO_4_^2−^ by SRB-6 at 5 °C, 10 °C, 20 °C, and 30 °C were 10.21%, 26.34%, 73.11%, and 88.86%, respectively.

### 2.6. Research on SRB Gel Particles

#### 2.6.1. Physical and Chemical Characteristic Analysis

**Figure 8:**
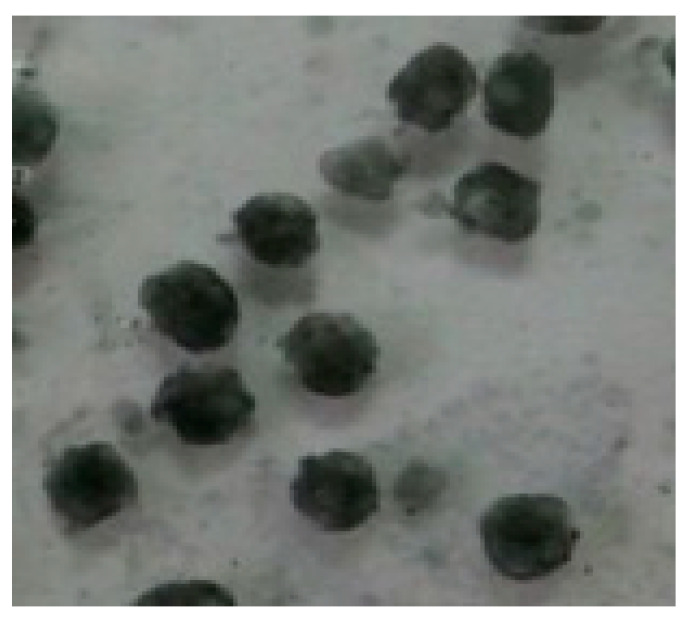
Morphological observation of SRB strains under a microscope.

The surface of the SRB gel sphere was smooth, black, and bright, exhibiting a granular structure that is nearly spherical or ellipsoidal (Figure 8). After being stored at 4 °C for 8 h, the SRB gel sphere underwent slight deformation due to minor water loss, causing a slight reduction in volume and a shape that remained close to spherical. Further inspection revealed that the SRB gel spheres had a diameter of approximately 1.5–2.5 mm and surface holes and contained elements such as carbon (C), oxygen (O), nitrogen (N), phosphorus (P), and sulfur (S).

The authors note that the SRB gel spheres exhibited minor deformation following storage, yet their reusability is a pivotal consideration for practical applications. For repeated use, it was essential to assess the structural integrity and functional performance of the SRB gel spheres through multiple cycles of sulfate reduction and recovery. To ensure the reusability of the SRB gel spheres, several factors had to be evaluated. Firstly, the spheres must maintain their shape and avoid significant degradation or deformation after each use. Secondly, their sulfate reduction capability must remain consistent across multiple cycles. Thirdly, the presence of essential elements (C, O, N, P, and S) must not diminish significantly. Finally, the gel spheres’ ability to retain water and prevent excessive drying out during storage must be monitored to maintain their structural and functional integrity.

**Figure 9:**
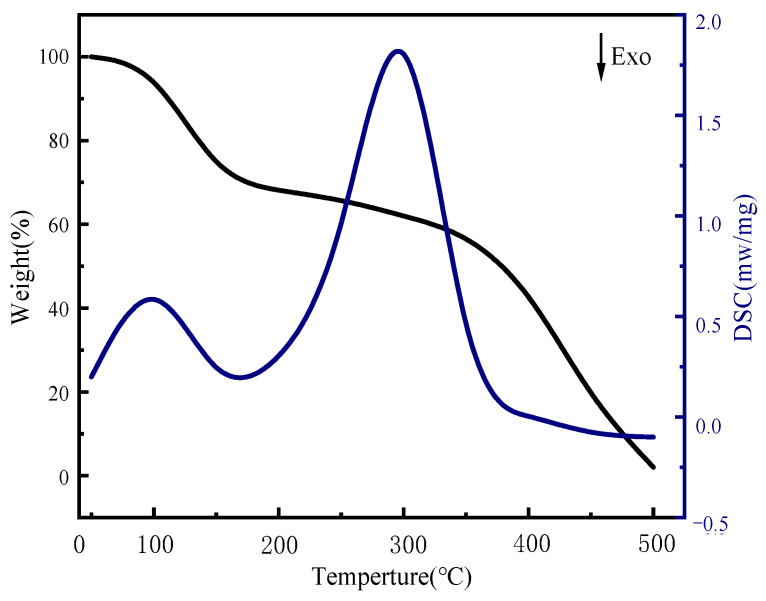
DSC-TGA of SRB gel spheres.

This paper presents the results of DSC-TGA of sulfate-reducing bacteria gel spheres, as illustrated in Figure 9. The provided DSC-TGA analysis offers valuable insights into the thermal properties and stability of sulfate-reducing bacteria (SRB) gel spheres. The TGA curve indicates that the SRB gel spheres maintained their weight stability up to approximately 100 °C, which is indicative of a low moisture content. Between 100 °C and 200 °C, slight weight loss accompanied by an endothermic peak in the DSC curve suggests the release of adsorbed water and low molecular weight volatiles. The most significant thermal event occurred between 200 °C and 400 °C, where major weight loss was accompanied by a pronounced endothermic peak, indicating the thermal degradation of organic components, including the bacterial biomass and gel matrix. Following decomposition, beyond 400 °C, the weight stabilized, and a minor exothermic peak suggests secondary thermal events such as recrystallization or further chemical transformations of the residual material. This comprehensive thermal analysis is essential for understanding the thermal behavior and stability of SRB gel spheres and demonstrates that this potent SRB gel sphere can slowly dissolve in an aqueous medium, effectively controlling the growth of any microorganisms in an aquatic environment.

#### 2.6.2. Conductivity

**Figure 10:**
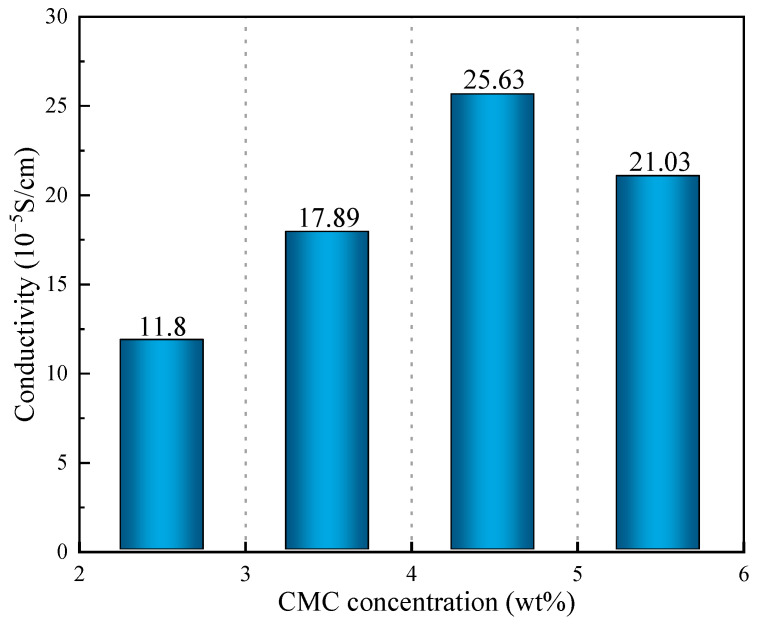
Conductivity of CMC solutions at various concentrations (wt%).

According to the above results (Figure 10), the conductivity of the SRB gel balls first increased and the decreased with the increase in CMC density, and when the CMC density was 4.5%, the conductivity value reached its maximum (2.563 × 10^−4^ s/cm). When the concentration of CMC was low, the network structure formed by the gel balls was loose and sparse, which is not conducive to the attachment of PAn, thus making it difficult to form a conductive channel. Consequently, the gel balls exhibited low conductivity. When the CMC density was high, a coordinated reaction between the CMC and Fe^3+^ in the gel balls would occur, resulting in blockage of the channel and hindering the entry of PAn, thereby reducing the conductivity.

**Figure 11:**
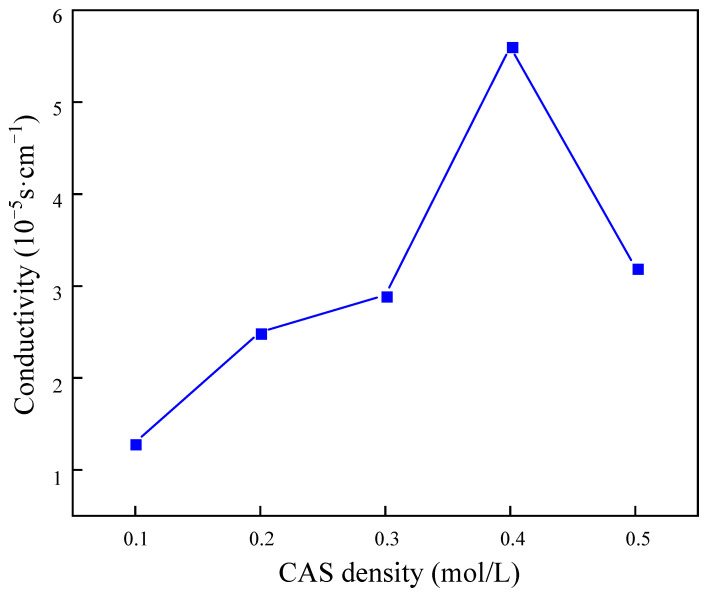
Influence of sodium benzenesulfonate concentration on electrical conductivity of gel spheres.

The results presented in Figure 11 demonstrate that the macromolecules in the gel balls intertwined with each other to form a uniform and fine pore structure. As the concentration of BSNa increased, the cross-linked network exhibited a tendency towards greater density. Following the analysis of the rate of sulfate removal by the gel balls at varying densities of BSNa, it was determined that at a BSNa density of 0.4 mol/L, the highest conductivity was 5.62 × 10^−3^ s/cm. However, an excessively high density of BSNa may result in damage to the structure of PAn, potentially leading to a reduction in conductivity due to the non-conductive nature of BSNa.

#### 2.6.3. Water Loss Rate and Cross-Linking Time

**Figure 12:**
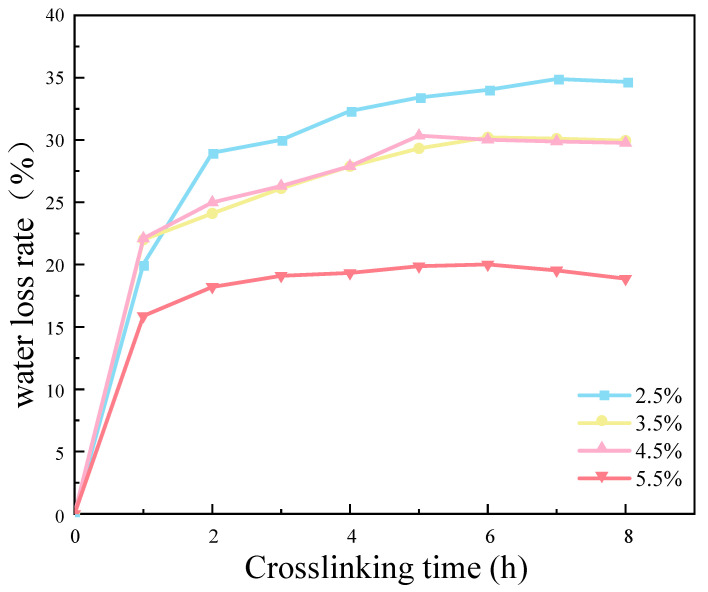
Effect of cross-linking time and sodium benzenesulfonate concentration on weight loss rate of gel spheres.

The results demonstrate that an increase in CMC density was associated with a reduction in the water loss rate of the SRB gel balls. This occurred primarily within the first hour following the cross-linking process (Figure 12). The experimental results demonstrate that the water loss rate of the CMC density of 5.5% was significantly lower than those of the other three densities of samples. This was primarily due to the low water content of the gel balls themselves, which increased with an increase in the CMC density. Additionally, CMC is a hydrophilic substance which reduces the free water lost during the water binding process. Consequently, the water loss rate of the gel balls was lowest when the CMC density was 5.5%. Figure 12 illustrates that the degree of cross-linking of the gel balls was essentially saturated at 8 h, which ensured that the gel balls had a certain hardness. Consequently, the cross-linking time to ensure a complete and efficient reaction was determined to be 9 h.

#### 2.6.4. The Removal Rate of Sulfate Ions in the Treatment of AMD

In light of the aforementioned determination experiments, SRB-6 was selected as the experimental bacterium, and the requisite environment was established. This environment was defined by the following parameters: pH = 6, C/S = 1.5, T = 30 °C, CMC = 4.5%, BSNa = 0.4 mol/L, and a cross-linking time of 9 h. The sulfate-reducing bacteria gel particles prepared under the conditions of CMC = 4.5% and BSNa = 0.4 mol/L were reacted with the AMD. The sulfate radical removal rate of the AMD by the immobilized sulfate-reducing bacteria gel particles was found to reach 91.6%, with a correspondingly positive treatment effect.

### 2.7. Microbial Abundance Analysis

**Figure 13:**
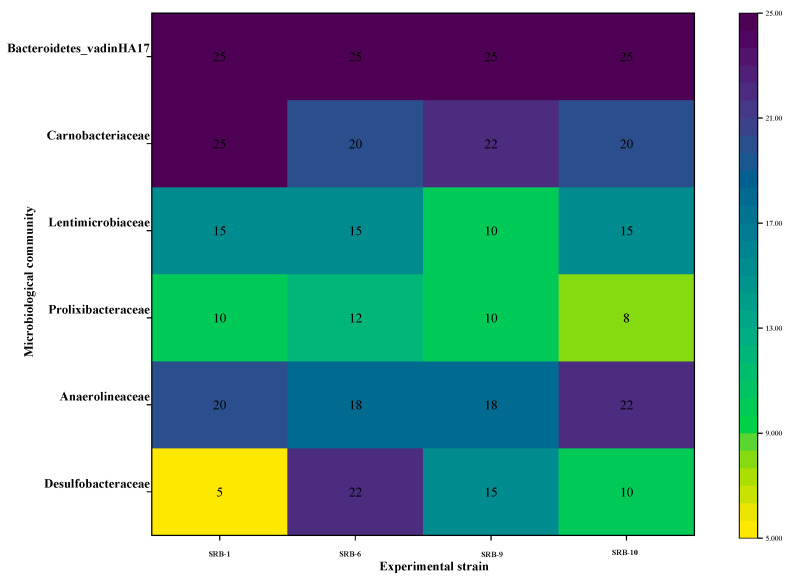
Heat map of microbial community composition across different experimental strains.

Figure 13 illustrates the prevalence of several microbial taxa across different experimental strains. The color of the heatmap indicates the level of microbial abundance. The abundance of *Bacteroides_vadinHA17* remained at 25% in all of the experimental strains, indicating that this microbial taxon maintains a high level of stability under different experimental conditions. *Carnobacteriaceae* demonstrated greater abundance (22%) under the SRB-6 experimental conditions, in comparison with a relatively lower abundance (20%) under other conditions. This may be indicative of more favorable conditions for this taxon in SRB-6. *Lentimicrobiaceae* exhibited the lowest abundance (10%) under the SRB-9 experimental conditions, while it exhibited relatively high abundance (15%) under the other conditions. The lowest abundance of *Prolixibacteraceae* was observed in the SRB-9 experimental conditions (8%), with an increase observed in the SRB-6 conditions (12%). This may indicate that different conditions have significantly different effects on the growth of this microorganism. *Anaerolineaceae* showed the greatest abundance (22%) under the SRB-9 experimental conditions and slightly lower abundance (18–20%) under the other conditions. This suggests that the SRB-9 conditions in particular may have facilitated the growth of this taxon. *Desulfobacteraceae* also showed the highest abundance (22%) under the SRB-6 experimental conditions and the lowest abundance (5% and 10%) under the SRB-1 and SRB-10 experimental conditions, respectively, reflecting the different effects of different conditions on this group of microorganisms. *Desulfobacteraceae* is a key microbial group representing sulfate-reducing bacteria. These microorganisms play an important role in wastewater treatment and ecosystems and are effective in AMD degradation and sulfur cycling. *Desulfobacteraceae* showed the greatest abundance (22%) under the SRB-6 experimental conditions. This suggests that these conditions may have provided a more suitable environment to promote the growth of sulfate-reducing bacteria. Possible facilitating factors include nutrient availability, the environmental pH level, temperature, or other biochemical conditions. This may indicate that the growth of this microorganism is significantly influenced by different conditions. *Anaerolineaceae* exhibited the greatest abundance (22%) under the SRB-9 experimental conditions, with a slightly lower (18–20%) abundance observed under the other conditions. This indicates that the SRB-9 conditions may have been particularly conducive to the growth of this taxon. *Desulfobacteraceae* also exhibited the greatest abundance (22%) under the SRB-6 experimental conditions and the lowest (5% and 10%) abundance under the SRB-1 and SRB-10 experimental conditions, respectively, reflecting the differential effects of the various conditions on this group of microorganisms. *Desulfobacteraceae* represents a key microbial group, comprising sulfate-reducing bacteria. These microorganisms play a pivotal role in wastewater treatment and ecosystems and are effective in AMD degradation and sulfur cycling. *Desulfobacteraceae* exhibited the greatest abundance (22%) under the SRB-6 experimental conditions. This indicates that the condition may have provided a more conducive environment for the proliferation of sulfate-reducing bacteria. It is possible that the facilitating factors include the availability of nutrients, the environmental pH level, temperature, or other biochemical conditions.

## 3. Materials and Methods

### 3.1. Materials

Yeast extract, sodium lactate, trisodium citrate, resazurim, ascorbic acid, MgSO_4_·7H_2_O, Na_2_SO_4_, sodium thioglycolate, sodium alginate (SA), carboxymethyl cellulose (CMC), aniline (AN), activated carbon (GAC), graphene oxide (GO), sodium benzene sulfonate (BSNa), and CaCl_2_ were used in this study. All experimental drugs were analytically pure, and the experimental water was deionized water.

### 3.2. Preparation of the Materials

It was necessary to collect sludge and contaminated water, which was produced in an anaerobic environment at the water gushing point of the AMD and in nearby rivers and wetlands that had been polluted by AMD, as well as in the pretreatment process of AMD. A total of 20–30 samples were collected from 8–10 sites. The samples were immediately placed into pre-sterilized glassware, sealed to ensure as anaerobic an environment was maintained as possible, and then stored in a refrigerator at a temperature of −20 °C for subsequent analysis.

### 3.3. Experimental Methods

In this experiment, sludge was anaerobically incubated in a constant temperature shaker at 35 °C and 150 r/min for 4–6 days. This was followed by inoculation into modified Postgate medium, which was repeated five times to select the best-growing SRB suspensions. The suspensions were then incubated by the dilution coating layered sandwich method, with the objective of isolating small, individual black colonies of various morphologies, sizes, and colors. Finally, the suspensions were anaerobically cultured. Three distinct gel particles were prepared at a solid-to-liquid ratio (M/V) of 10% for the treatment of acid mine wastewater. The adsorption of sulphate was then investigated under static conditions, with three sets of parallel experiments set up for each group.

### 3.4. Analytical Method

Determination of the pH level was carried out using a pH meter (PHS-3C; Leici, Shanghai, China). The mass concentration of sulfate was determined through ion chromatography using an ion chromatograph (ICS-5000; Thermo Scientific, Waltham, MA, USA).

DSC (SeikoDSC6100, Chiba, Japan) was used to determine the thermal transition properties of resistant dextrins. Each sample (3 mg, dry base) was moistened with distilled water (7 μL) in a DSC dish and equilibrated at 4 °C for 24 h prior to analysis. The DSC disc was heated at a rate of 5 °C/min in the range of 0–500 °C, with the empty disc as a reference. A thermal analysis system (EXSTAR-6000; Seiko, Chiba, Japan) was utilized.

This experiment adopted the model of the scanning electron microscope (SEM, HITACHISU8010; Hitachi, Japan) for electron microscopy (SEM) analysis. Scanning electron microscopy (SEM) employs the use of high-energy electron beams focused into extremely fine dots which are then scanned point by point on the surface of a sample. This process allows for the excitation and collection of a diverse range of physical information. The surface structure features of the tested sample are revealed by the process of receiving, magnifying, and displaying the aforementioned information [27,28,29].

## 4. Conclusions

This study demonstrates that sulfate-reducing bacteria (SRB) gel particles are an effective type of treatment for acid mine drainage (AMD). The SRB strains were observed to demonstrate high sulfate removal efficiency under optimal conditions. In particular, the SRB gel particles, when prepared with sodium alginate (SA) and carboxymethyl cellulose (CMC), demonstrated sulfate removal rates of up to 91.6% at a pH of six, C/S ratio of 1.5, temperature of 30 °C, CMC concentration of 4.5%, and BSNa concentration of 0.4 mol/L. These findings illustrate the potential of SRB gel particles for practical applications in the bioremediation of AMD.

Nevertheless, it is essential to address several limitations in future research. Firstly, the long-term stability and reusability of SRB gel particles must be thoroughly evaluated under a range of environmental conditions. The slight deformation observed in the SRB gel spheres after storage indicates the potential for structural integrity to be compromised over extended periods and repeated use. Furthermore, the scalability of this treatment method must be evaluated to ascertain its suitability for large-scale industrial applications.

Future research should also investigate the genetic and metabolic pathways of SRB in order to enhance their sulfate reduction efficiency. An investigation into the interactions between SRB and other microbial communities in AMD-affected environments could provide insights into the optimization of treatment conditions. Moreover, the integration of SRB gel particles with other treatment technologies could enhance the overall effectiveness and efficiency of AMD remediation.

In conclusion, while the SRB gel particles showed promise for the treatment of age-related macular degeneration (AMD), it is essential that the limitations and challenges outlined in this study be addressed through future research and development if their full potential for practical applications is to be realized.

## Figures and Tables

**Table 1:** Preliminary screening of SRB strains.

Strain Name	Fermentation pH Value	Blackening Time of Medium
Strain 1	8.71	27
Strain 2	8.60	40
Strain 3	8.03	50
Strain 4	7.89	48
Strain 5	8.52	48
Strain 6	8.88	24
Strain 7	8.60	29
Strain 8	8.21	42
Strain 9	8.74	25
Strain 10	8.82	26

**Table 2:** Analysis of physiological and biochemical characteristics of SRB strains after preliminary screening.

Strain Name	Gram Stain	Electron Receptor Experiment	Gelatin Liquification Experiments	V-P Test	Sugar Fermentation Experiment			Electron Acceptor Utilization Experiment	
					Glucose	Sucrose	Lactose	Na_2_SO_4_	Sulfur
SRB-1	-	+	+	-	-	+	+	+	+
SRB-6	-	+	+	-	-	+	+	+	+
SRB-9	-	+	-	-	-	+	+	+	+
SRB-10	-	+	+	-	-	+	+	+	+

+ = Gram-positive or available; - = Gram-negative or unavailable.

## Data Availability

The experimental data used to support the findings of this study are included in the article.

## References

[1] Yang Y., Li B., Li T., Liu P., Zhang B.B., Che L.L. (2023). A review of treatment technologies for acid mine drainage and sustainability assessment. J. Water Process Eng..

[2] Liu Y., Xie X.M., Wang S., Hu S.M., Wei L.Z., Wu Q.H., Luo D.G., Xiao T.F. (2023). Hydrogeochemical evolution of groundwater impacted by acid mine drainage (AMD) from polymetallic mining areas (South China). J. Contam. Hydrol..

[3] Luo J.Z., Cai Y.Y. (2024). Mechanism of the pore and molecular structure evolution of coal exposed to acid mine drainage (AMD). Sci. Total Environ..

[4] Ngole-Jeme V.M., Ndava J. (2023). The implications of AMD induced acidity, high metal concentrations and ochre precipitation on aquatic organisms. Pol. J. Environ. Stud..

[5] Jiao Y.N., Zhang C.H., Su P.D., Tang Y.H., Huang Z.P., Ma T. (2023). A review of acid mine drainage: Formation mechanism, treatment technology, typical engineering cases and resource utilization. Process Saf. Environ..

[6] Mosai A.K., Ndlovu G., Tutu H. (2024). Improving acid mine drainage treatment by combining treatment technologies: A review. Sci. Total Environ..

[7] Shi W.Z., Zhao C.H., Liang Y.P., Han Z.T., Xie H., Tang C.L. (2022). Genetic mechanism analysis of low Ca/Mg value of acid goaf water in coal mine drainage. Carsologica Sin..

[8] Liang Y.P., Shen H.Y., Gao X.B. (2022). Review of research progress of karst groundwater in northern China. Bull. Geol. Sci. Technol..

[9] Zhao C.H., Liang Y.P., Wang Z.H., Tang C.L., Shen H.Y. (2023). Dynamic characteristics and evolution mechanism of “goaf water” of coal mine in Shandi River Basin, Yangquan, Shanxi Province and its environmental effects on Niangziguan Spring Area. Geol. China.

[10] Zhang H.G., Li M., Yang Z.Q., Sun Y.Q., Yan J., Chen D.Y., Chen Y.H. (2017). Isolation of a non-traditional sulfate reducing-bacteria *Citrobacter freundii* sp. and bioremoval of thallium and sulphate. Ecol. Eng..

[11] Pape P.L., Battaglia-Brunet F., Parmentier M., Joulian C., Gassaud C., Fernandez-Rojo L., Guigner J.M., Ikogou M., Stetten L. (2017). Complete removal of arsenic and zinc from a heavily contaminated acid mine drainage via an indigenous SRB consortium. J. Hazard. Mater..

[12] Yan J., Zhong K.Q., Wang S.J., Chen Z.X., Hu H.S., Jian Z.Y., Wen H.J., Zhang H.G. (2018). Carbon metabolism and sulfate respiration by a non-conventional *Citrobacter freundii* strain SR10 with potential application in removal of metals and metalloids. Int. Biodeter. Biodegr..

[13] Miao Y.H., Qi S.Y., Chen J., Wang J., Tian B.Y., Xin B.P. (2021). Application of sulphate reducing bacteria in acid mine drainage treatment: A review. Appl. Chem. Ind..

[14] Zhang Z., Zhang C.H., Yang Y., Zhang Z.W., Tang Y.H., Su P.D., Lin Z.W. (2022). A review of sulfate-reducing bacteria: Metabolism, influencing factors and application in wastewater treatment. J. Clean. Prod..

[15] Guo X.Y., Hu Z.Y., Dong Y.G., Fu S.O., Li Y. (2022). Study of the preparation of Maifan stone and SRB immobilized particles and their effect on treatment of acid mine drainage. RSC Adv..

[16] Novair S.B., Atigh Z.B.Q., Lajayer B.A., Shu W.X., Price G.W. (2024). The role of sulphate-reducing bacteria (SRB) in bioremediation of sulphate-rich wastewater: Focus on the source of electron donors. Process Saf. Environ..

[17] Hwang S.K., Jho E.H. (2018). Heavy metal and sulfate removal from sulfate-rich synthetic mine drainages using sulfate reducing bacteria. Sci. Total Environ..

[18] Magowo W.E., Sheridan C., Rumbold K. (2020). Global co-occurrence of acid mine drainage and organic rich industrial and domestic effluent: Biological sulfate reduction as a co-treatment-option. J. Water Process Eng..

[19] Chen X.Y., Feng J., Wang H.M., Liu D., Dou Y.F., Zhang J.H., Ma L.Y. (2022). Isolation and identification of sulfate-reducing bacteria in goaf water in Yangquan of Shanxi Province and domestication for the reduction. Acta Microbiol. Sin..

[20] Diao C.Y., Ye W.Z., Yan J., Hao T.W., Huang L., Chen Y.H., Long J.Y., Xiao T.F., Zhang H.G. (2023). Application of microbial sulfate-reduction process for sulfate-laden wastewater treatment: A review. J. Water Process Eng..

[21] Zhang H.G., Li M., Pang B., Wu Y.J., Sun Y.Q., Chen D.Y., Chen Y.H. (2017). Bioremoval of Tl (I) by PVA-immobilized Sulfate-Reducing Bacteria. Pol. J. Environ. Stud..

[22] Zhang H.G., Li M., Li H.S., Li M., Luo D.G., Chen Y.H., Chen D.Y., Luo H.L., Chen Z.X., Li K.K. (2018). Immobilizing metal-resistant sulfate-reducing bacteria for cadmium removal from aqueous solutions. Pol. J. Environ. Stud..

[23] Xin Z.J., Wang X.Y., Li L., Li Y., Deng M., Yao Z. (2024). Components optimization of immobilized SRB-embeded particles and study on the treatment effect of sulfate-containing wastewater. Ind. Water Treat..

[24] Zhang L., Chen Y., Zhang L., Song R.K., Wang Q., Liu C.J., Mu Y.D. (2024). Preparation of ZrO_2_/γ-Al_2_O_3_ albumen type catalyst and its catalytic performance for carbonyl sulfide hydrolysis. Appl. Phys. A.

[25] Zhang L., Wang Q., Zhao H.C., Song R.K., Chen Y., Liu C.J., Han Z.K. (2024). Synthesis and surface strengthening modification of Silica aerogel from fly ash. Materials.

[26] Zhang L., Song R.K., Jia Y., Zou Z.R., Chen Y., Wang Q. (2024). Purification of quinoline insolubles in heavy coal tar and preparation of meso-carbon microbeads by catalytic polycondensation. Materials.

[27] Yuan Y.G., Leng C.P., Zhou Y.L., Yuan Y., Niu Y.X., Xu R.Y., Zhong H.Y., Li F.P., Zhou H.X., Wang H. (2023). Impact of separate concentrations of polyethylene microplastics on the ability of pollutants removal during the operation of constructed wetland-microbial fuel cell. J. Environ. Manag..

[28] Xu R.Y., Yang Z.N., Niu Y.X., Xu D., Wang J., Han J.L., Wang H. (2022). Removal of microplastics and attached heavy metals from secondary effluent of wastewater treatment plant using interpenetrating bipolar plate electrocoagulation. Sep. Purif. Technol..

[29] Wang H., Zhong Y.L., Bo G.Z. (2018). Existing forms and changes of nitrogen inside of horizontal subsurface constructed wetlands. Environ. Sci. Pollut. Res..

